# Novel Polycyclic Fused Amide Derivatives: Properties and Applications for Sky-blue Electroluminescent Devices

**DOI:** 10.3390/molecules27165181

**Published:** 2022-08-14

**Authors:** Guo-Xi Yang, Deng-Hui Liu, Si-Min Jiang, Zhi-Hai Yang, Zi-Jian Chen, Wei-Dong Qiu, Yi-Yang Gan, Kun-Kun Liu, De-Li Li, Shi-Jian Su

**Affiliations:** State Key Laboratory of Luminescent Materials and Devices, Institute of Polymer Optoelectronic Materials and Devices, South China University of Technology, Wushan Road 381, Guangzhou 510640, China

**Keywords:** OLEDs, polycyclic fused amide, electron acceptor unit, blue emission

## Abstract

Aromatic imide derivatives play a critical role in boosting the electroluminescent (EL) performance of organic light-emitting diodes (OLEDs). However, the majority of aromatic imide-based materials are limited to long wavelength emission OLEDs rather than blue emissions due to their strong electron-withdrawing characteristics. Herein, two novel polycyclic fused amide units were reported as electron acceptor to be combined with either a tetramethylcarbazole or acridine donor via a phenyl linker to generate four conventional fluorescence blue emitters of **BBI-4MeCz**, **BBI-DMAC**, **BSQ-4MeCz** and **BSQ-DMAC** for the first time. **BSQ-4MeCz** and **BSQ-DMAC** based on a BSQ unit exhibited higher thermal stability and photoluminescence quantum yields than **BBI-4MeCz** and **BBI-DMAC** based on a BBI unit due to their more planar acceptor structure. The intermolecular interactions that exist in the BSQ series materials effectively inhibit the molecular rotation and configuration relaxation, and thus allow for blue-shifted emissions. Blue OLED devices were constructed with the developed materials as emitters, and the effects of both the structure of the polycyclic fused amide acceptor and the electron donor on the EL performance were clarified. Consequently, a sky-blue OLED device based on **BSQ-DMAC** was created, with a high maximum external quantum efficiency of 4.94% and a maximum luminance of 7761 cd m^−2^.

## 1. Introduction

As the next generation of display and lighting technology, the development of organic light-emitting diodes (OLEDs) has been undergoing a marvelous revolution since the first double-layered electroluminescent (EL) device was discovered by Tang and Van Slyke in 1987 [1]. Blue emission materials are of particular attention thanks to their variety of merits for application in OLEDs [2,3,4]. Firstly, as one of the three-primarily colors, the blue emitter is an indispensable component of full-color flat panel displays and solid-state lighting [5]. Secondly, the display structure can be greatly simplified by energy transfer from blue emissive materials to long-wavelength emission materials [6]. Moreover, blue emission materials can fully meet the obligation of energy-saving and a wide color gamut for commercial applications [7]. Nevertheless, the development of blue fluorophores is far from satisfactory due to their tough molecular designs in contrast to green and red fluorophores [8]. To date, the majority of works based on thermally activated delayed fluorescence (TADF) and “hot exciton” emitters have explored, but only a few can truthfully exhibit excellent stability for commercial blue OLEDs [9,10,11].

As of yet, conventional fluorophores are still the best choices for stable blue emission. However, molecular design units mainly focus on anthracene, pyrene, fluorene and their derivatives [12,13,14]. Therefore, the design and synthesis of novel building blocks for constructing highly efficient and stable conventional OLED devices are of particular importance. Recently, aromatic imide-based materials aroused huge attention thanks to several advantages such as their high photoluminescence quantum yields (PLQYs), rigid planar structure, excellent photochemical stability and strong electron-withdrawing characteristics [15,16,17]. For instance, in 2018, Yang and co-workers explored two novel efficient imide-based emitters using 1,8-naphthalimide and rigid acridine as the acceptor and donor, respectively [18]. The two reported emitters showed high PLQYs and preferentially horizontal emitting dipole orientation characteristics with external quantum efficiencies (EQEs) approaching 30%. Bin et al. designed and synthesized two materials with a new acceptor fused by pyrazine and maleimide in 2021 [19]. By elaborating the device structure, the orange-red OLED exhibited high EL performance with an EQE_max_ of 26.0% and a remarkably low efficiency roll-off. They demonstrated that the imide unit is an effective candidate for building highly efficient OLEDs. Nonetheless, a lot of imide-based materials utilized for OLED applications show red-shifted emissions upon straight conjugation with acceptor units [20,21,22]. This results in an alteration in the spectral pattern and harshly breaks color purity. Hence, it is still challenging to develop controllable acceptor units for achieving high blue EL performance [23].

In this work, we reported four donor-bridge-acceptor (D-π-A) blue emissive materials, 13-(4-(1,3,6,8-tetramethyl-9*H*-carbazol-9-yl)phenyl)benzo[*cd*]benzo [5,6][1,2]thiazino[2,3-*a*]indole 8,8-dioxide (**BBI-4MeCz**), 13-(4-(9,9-dimethylacridin-10(9*H*)-yl)phenyl)benzo[*cd*]benzo[5,6][1,2]thiazino[2,3-*a*]indole 8,8-dioxide (**BBI-DMAC**), 13-(4-(1,3,6,8-tetramethyl-9*H*-carbazol-9-yl)phenyl)-8*H*-benzo[3,4]indolo[1,2-*b*]isoquinolin-8-one (**BSQ-4MeCz**) and 13-(4-(9,9-dimethylacridin-10(9*H*)-yl)phenyl)-8*H*-benzo[3,4]indolo[1,2-*b*]isoquinolin-8-one (**BSQ-DMAC**), based on novel polycyclic fused amide skeletons as electron acceptor units. Compared with conventional imide units, the lack of carbonyl groups in the amide unit efficiently decreases the electron-withdrawing ability and thus controls intramolecular charge transfer (ICT) to maintain blue emissions. The polycyclic fused structure can provide high PLQYs, and the large torsional angle derived from the highly twisted structure provides high color purity by restricting unattractive intermolecular interactions. The photophysical properties of the four emitters were systematically investigated here. The four emitters exhibited similar photophysical behavior with the corresponding acceptor units, indicating the predominance of novel amide skeletons. Additionally, the BSQ derivatives showed greater blue emissions than the BBI derivatives for the more inflexible molecular planarity structures. Blue OLED devices based on the use of the as-synthesized materials as emitters were fabricated with a low turn-on voltage (*V*_on_) and decent EL performance. Specifically, the devices based on **BBI-4MeCz**, **BBI-DMAC**, **BSQ-4MeCz** and **BSQ-DMAC** as emitters achieved EQEs of 2.95%, 2.97%, 4.13% and 4.45%, with emission peaks of 494 nm for the BBI derivatives and 488 nm for the BSQ derivatives, respectively. Furthermore, the best EL performance based on the use of **BSQ-DMAC** as an emitter was obtained by elaborating the dopant concentration, with an extremely low *V*_on_ of 2.8 V and an EQE_max_ of 4.94% at an EL peak of 492 nm. This work might provide effective suggestions for the future exploration of blue emission materials.

## 2. Results and discussion

### 2.1. Synthesis and Single crystals

**Scheme 1:**
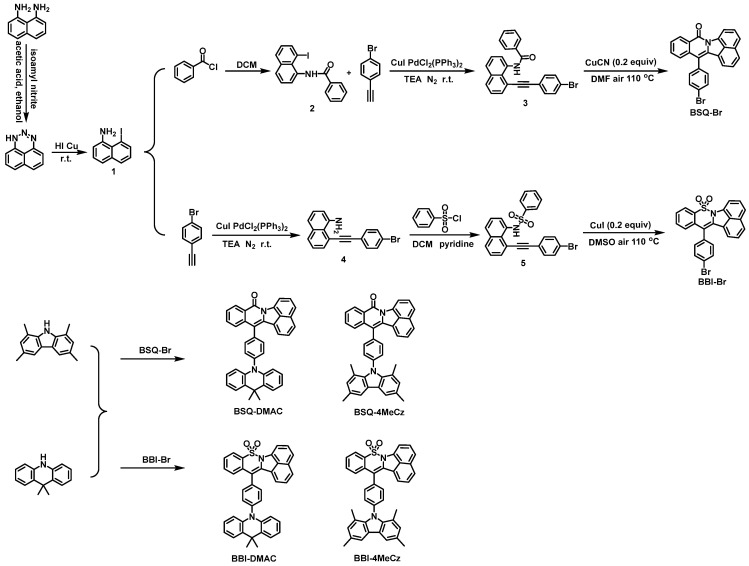
The synthetic routes of the target compounds.

All reagents and solvents were purchased from commercial sources and used without further purification. The synthetic routes of the four compounds are outlined in Scheme 1. The detailed synthesis information is summarized in the Supporting Information. The compounds BSQ-Br and BBI-Br were synthesized by copper-catalyzed cyclization reactions, according to previously reported works [24,25,26,27,28]. Then, the target materials were successfully acquired through the reaction of transition metal-catalyzed N-C bond formation between the intermediate compounds [29]. Afterward, the products were purified by silica column chromatography and temperature-gradient sublimation with ^1^H/^13^C NMR, MALDI-TOF mass spectrometry (Figure S1–S9, Supporting Information) and single crystal X-ray diffraction (XRD).

**Figure 1:**
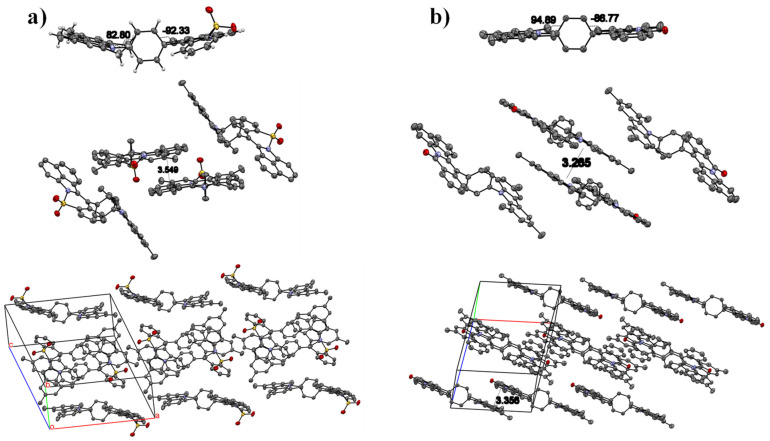
Single crystal structures and packing modes of (**a**) BBI-4MeCz and (**b**) BSQ-4MeCz.

Single crystals of **BBI-4MeCz** and **BSQ-4MeCz** (CCDC 2201090 and 2201089) were successfully obtained through slow diffusion from a mixed solution of chloroform and methanol. The crystal structures and molecular packing are presented in Figure 1. Obviously, the minimum unit cell is formed by four monomers for two crystals. Both **BBI-4MeCz** and **BSQ-4MeCz** exhibited twisted structures with a torsion angle between the central phenyl ring and D/A unit of almost 90°, which matched well with the quantum computation (vide infra). However, compared with **BSQ-4MeCz**, **BBI-4MeCz** displayed an arc-shaped single crystal structure due to the two oxygen atoms connected to the sulfur atom. This non-planar structure led to a broken conjugation and thus resulted in a low PLQY. With the introduction of a bulky tetramethylcarbazole unit into the BBI acceptor unit, only weak π-π stacking existed between two molecules, with a distance of 3.549 Å. On the contrary, the **BSQ-4MeCz** crystal displayed more intermolecular π-π stacking interactions (3.265 and 3.356 Å) between the monomer skeletons. The relatively stronger intermolecular interactions in **the BSQ-4MeCz** can improve molecular rigidity and significantly restrain the nonradiative decay, thus enhancing the PLQY and charge transport capacity. These results demonstrate that BSQ-based emitters can perform better than BBI-based emitters in OLED devices.

### 2.2. Theoretical Calculations

**Figure 2:**
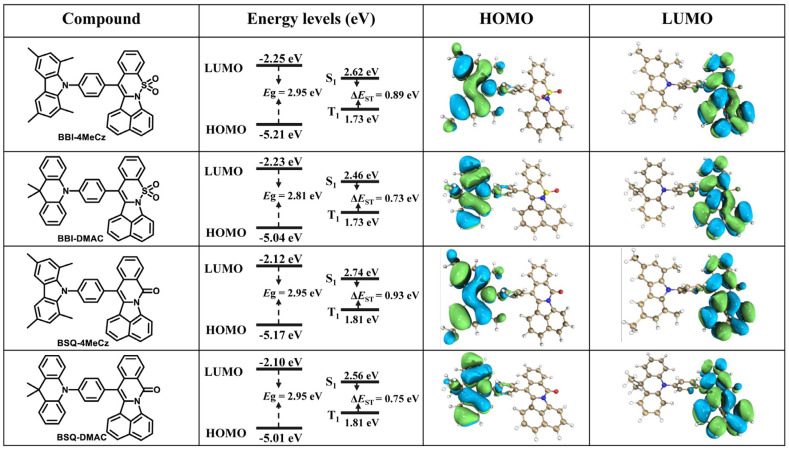
Molecular structures, calculated energy levels of HOMO, LUMO, S_1_ and T_1_, and the calculated HOMO and LUMO distributions based on b3lyp/6-31g(d,p).

Density functional theory (DFT) was implemented based on the Gaussian 16 program package at a B3LYP/6-31g(d,p) level to analyze the structure-property relationships. As shown in Figure S10, the optimized ground state geometries of all four emitters exhibited nearly orthogonal structures. The dihedral angles between the benzene ring and the D/A units were (86.8°, −98.7°), (88.7°, −99.3°), (90.3°, −91.3°) and (88.5°, −100.0°) in the **BBI-4MeCz**, **BBI-DMAC**, **BSQ-4MeCz** and **BSQ-DMAC**, respectively. Owing to the highly distorted structure, the intermolecular conjugation and electron exchange were efficiently suppressed to avoid exciton quenching and to give blue emissions [10]. The distributions of the highest occupied molecular orbitals (HOMOs) and the lowest unoccupied molecular orbitals (LUMOs) of the four emitters were similar (Figure 2). In general, the HOMO was distributed in the carbazole or acridan donor units and the LUMO was located on the acceptor moieties.

Moreover, natural transition orbitals (NTOs) were also investigated with time-dependent density functional theory (TD-DFT). As shown in Figure S11 and S12, all emitters exhibited similar distributions of “holes” and “particles”. Specifically, the “holes” of the lowest singlet excited states (S_1_) were mainly distributed on the electron-donating carbazole or acridine units, while the “particles” were separated in the acceptor moieties. In the lowest triplet excited states (T_1_), their “holes” and “particles” were clearly located at the acceptor cores, representing the locally excited (LE) features. The S_1_ and T_1_ energy levels itemized in Table 1 were also calculated by TD-DFT. The S_1_ energy levels of the **BBI-4MeCz**, **BBI-DMAC**, **BSQ-4MeCz** and **BSQ-DMAC** were 2.62, 2.46, 2.74 and 2.56 eV, respectively, and the T_1_ energy levels were calculated as being 1.73, 1.73, 1.81 and 1.81 eV, respectively. No matter whether tetramethylcarbazole or acridan was combined with a BBI or BSQ unit, the T_1_ energy levels were unchanged for the BBI or BSQ-based materials, indicating that the triplet emission may have originated from the acceptor fragments. Besides this, the corresponding splitting energies (Δ*E*_ST_s) between the S_1_ and T_1_ were over 0.7 eV, suggesting that no TADF properties existed for these four emitters [30].

### 2.3. Thermal and Electrochemical Properties

The thermal stabilities of the four materials were verified by thermogravimetric analysis (TGA) and differential scanning calorimetry (DSC) under a nitrogen atmosphere. The TGA and DSC results of the target compounds are shown in Figure S13, with detailed data summarized in Table 1. All the materials presented high decomposition temperatures (*T*_d_s, weight loss of 5%) and the *T*_d_s for the **BBI-4MeCz**, **BBI-DMAC**, **BSQ-4MeCz** and **BSQ-DMAC** were 408, 419, 463 and 430 °C, respectively. The higher *T*_d_s of the **BSQ-4MeCz** and **BSQ-DMAC** were attributed to the more planar structure of the BSQ unit. Then, DSC measurements were employed to detect the glass transition temperatures (*T*_g_s) of these four emitters. Apparently, the *T*_g_s with endothermic steps was observed at 137, 131 and 126 °C for **BBI-4MeCz**, **BBI-DMAC** and **BSQ-DMAC**, respectively, whereas no obvious *T*_g_ was observed for **BSQ-4MeCz**. Generally, the outstanding thermal performance indicated that all the as-synthesized materials were thermodynamically stable during the fabrication process of the electroluminescent devices [31].

A photoelectron emission spectrometer was used to measure the frontier orbital energy levels. The related atmospheric ultraviolet photoelectron spectroscopies are shown in Figure S14 and the detailed data are listed in Table 1. The HOMOs were estimated to be −5.87, −5.77, −5.93 and −5.72 eV for the **BBI-4MeCz**, **BBI-DMAC**, **BSQ-4MeCz** and **BSQ-DMAC**, respectively. The shallower HOMO levels of the **BBI-DMAC** and **BSQ-DMAC** can be ascribed to the stronger electron-donating ability of DMAC. Simultaneously, the shallower HOMO energy was in favor of hole injection, especially for the **BSQ-DMAC**. According to the value of the optical bandgap (*E*_g_, calculated from the absorption edge), the corresponding LUMOs were estimated as −3.14, −3.04, −3.17 and −2.96 eV, respectively. Suitable orbital energy levels are a prerequisite for highly efficient electroluminescent devices.

### 2.4. Photophysical Properties

**Figure 3:**
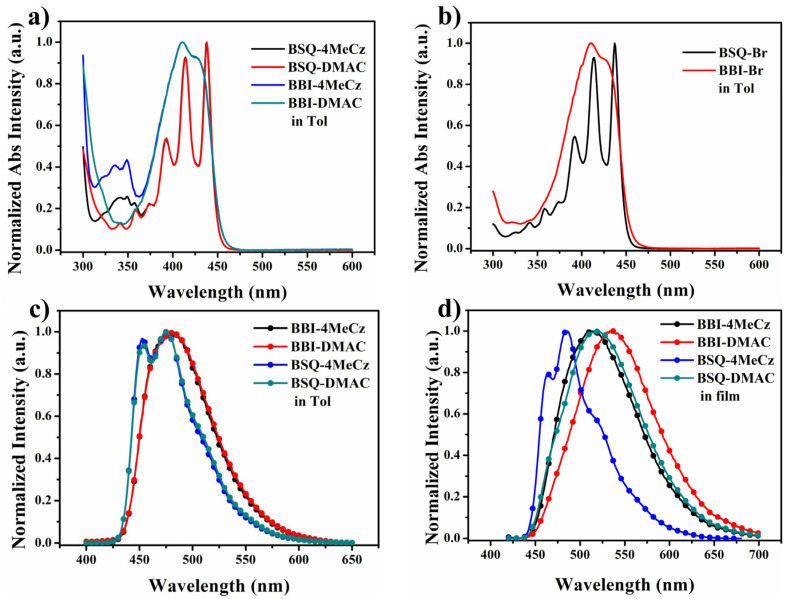
UV-vis absorption spectra of the (**a**) target compounds and (**b**) acceptor units in toluene solution. PL spectra of the four emitters in (**c**) toluene solution and (**d**) non-doped thin film formed by spin-coating.

The photophysical properties of the **BBI-4MeCz**, **BBI-DMAC**, **BSQ-4MeCz** and **BSQ-DMAC** in toluene solution and spin-coating film were tested by ultraviolet-visible (UV-vis) absorption and photoluminescence (PL) spectrometers. The related spectra are shown in Figure 3 and the detailed parameters are listed in Table 1. With the same acceptor moiety, analogous strong absorption characteristics were observed in the range of 390–450 nm for the **BSQ-4MeCz** and **BSQ-DMAC**, which should be ascribed to the π-π* transitions of the BSQ unit. Similarly, the **BBI-4MeCz** and **BBI-DMAC** also exhibited the same absorption band, with BBI-Br at the long wavelength for the common acceptor, while the short-wavelength absorption in the range of 300–370 nm for the **BSQ-4MeCz** and **BBI-4MeCz** should be attributed to the carbazole group. It is also worth mentioning that no obvious ICT character was observed, which is probably due to the twisted molecular structure (Figure 1). The *E*_g_s were calculated from the absorption edge to be 2.73, 2.73, 2.76 and 2.76 eV for **the BBI-4MeCz**, **BBI-DMAC**, **BSQ-4MeCz** and **BSQ-DMAC**, respectively, indicating their potential to realize sky-blue emissions.

For the PL spectra (Figure 3c,d), the emission peaks of the **BSQ-4MeCz** and **BSQ-DMAC** were located at 474 nm (toluene solution), blue-shifted by approximately 8 nm compared to that of the **BBI-4MeCz** and **BBI-DMAC** (482 and 481 nm), which were derived from a more twisted structure. The unchanged PL peaks for the introduction of electron-donating elements indicated the main control of the polycyclic fused amide frames. In the film, the emission peaks of the **BBI-4MeCz**, **BBI-DMAC**, **BSQ-4MeCz** and **BSQ-DMAC** were located at 512, 533, 482 and 517 nm. The shorter emission wavelength of the **BBI-4MeCz** and **BSQ-4MeCz** can be ascribed to the weak electron-donating ability of tetramethylcarbazole. Meanwhile, the small redshift seen with the **BBI-4MeCz** (30 nm) and **BSQ-4MeCz** (10 nm) in the solid state compared to that in toluene solution was also attributed to the steric hindrance of the tetramethylcarbazole.

**Figure 4:**
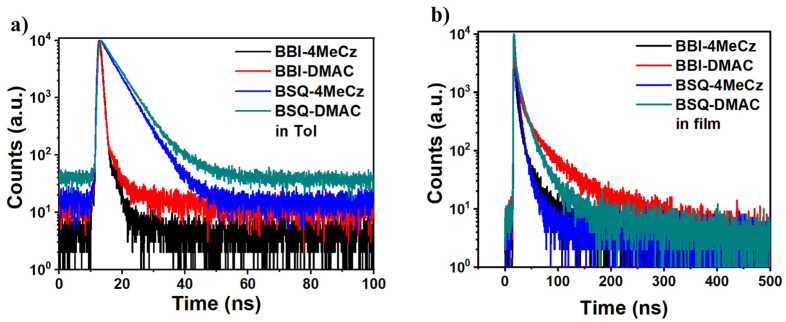
Transient PL decay spectra of the four emitters in (**a**) toluene solution and (**b**) non-doped thin film formed by spin-coating.

In addition, the PL properties of the four target materials and the acceptor moieties were also tested in different-polarity solvents; the related PL spectra are shown in Figure S15. Apparently, from low-polarity hexane to high-polarity acetonitrile, no obvious redshift was observed for the **BBI-4MeCz** and **BSQ-4MeCz**, indicating the LE characteristic in the excited state. However, an intense long-wavelength emission peak appeared in high-polarity solvents for the **BBI-DMAC** and **BSQ-DMAC**, suggesting CT features in the excited state. Besides this, the PLQYs in the solution conditions were detected to be 9.1%, 7.6%, 77.3% and 78.1% for the **BBI-4MeCz**, **BBI-DMAC**, **BSQ-4MeCz** and **BSQ-DMAC**, respectively, indicating that a higher EL performance is expected with the **BSQ-4MeCz** and **BSQ-DMAC**. The lower PLQYs for the **BBI-4MeCz** and **BBI-DMAC** were ascribed to the structural relaxation of the BBI unit. Transient PL decay spectra exhibited single exponential decay with a nanosecond lifetime in both toluene solution and thin film (Figure 4 and Table 1), suggesting the conventional fluorescence properties of these four emitters. The corresponding radiative rates (*Κ*_F_) were calculated, in which the **BSQ-4MeCz** possessed the highest *K*_F_ of 1.7 × 10^8^ s^−1^. Furthermore, the fluorescence and phosphorescence spectra at 77 K of these four emitters were also tested. In general, the low temperature fluorescence spectra exhibited hypochromatic shifts compared with those at room temperature, which can be attributed to suppressed molecular relaxation [32]. As shown in Figure S16, the PL spectra exhibited fine vibrational and structural bands, indicating the LE feature of all the emitters, which is perhaps due to the emissions coming from the donor or acceptor fragments.

### 2.5. Device Performance

**Figure 5:**
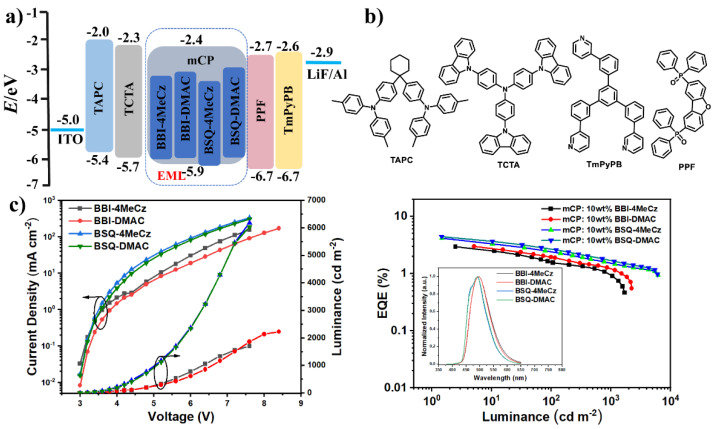
**(a**) Device architecture and energy level diagram; (**b**) Chemical structures of the related materials applied in devices; (**c**) *J-V-L* and EQE-L characteristics of the fabricated devices.

Considering the above-mentioned properties, blue emission devices using mCP (1,3-di(9*H*-carbazol-9-yl)benzene) as a host were fabricated with a configuration of ITO/TAPC (30 nm)/TCTA (20 nm)/mCP: 10 wt% dopant (20 nm)/PPF (10 nm)/TmPyPB (40 nm)/LiF (1 nm)/Al (100 nm). Here, ITO (indium tin oxide) and Al (aluminum) stand for anode and cathode. TAPC (4,4′-cyclohexylidene-bis(*N*,*N*-bis(4-methylphenyl)benzenamine]) and TCTA (4,4′,4″-tris(carbazol-9-yl)triphenylamine) are hole transporting and electron-blocking materials, respectively. PPF (2,8-bis(diphenylphosphoryl)dibenzo[*b,d*]furan), TmPyPB (1,3,5-tri[(3-pyridyl)-phen-3-yl]benzene) and LiF (lithium fluoride) serve as hole-blocking, electron transporting and electron injecting materials, respectively. By using **BBI-4MeCz**, **BBI-DMAC**, **BSQ-4MeCz** and **BSQ-DMAC** as emitter dopants, four OLED devices were obtained as devices A, B, C and D, respectively. The corresponding device architecture and energy diagram, related chemical structures of the materials used in the devices, current density-voltage-luminance (*J-V-L*) and EQE-luminance (EQE-L) characteristics are presented in Figure 5 with the key EL data listed in Table 2. As a result, the EL performance of the devices with **BSQ-4MeCz** and **BSQ-DMAC** as emitters were generally superior to those with **BBI-4MeCz** and **BBI-DMAC** in terms of EQE, emission peaks and luminance, etc. This can probably be ascribed to the higher PLQY and large *K*_F_ in the BSQ-based emitters. Specifically, devices A and B achieved an EQE_max_ of 2.95% and 2.97%, with the same emission peak of 494 nm, while the EQE_max_ of devices C and D was 4.13% and 4.45%, with an emission peak of 488 nm (Figure 5c). Apparently, no emission peaks other than those of the dopants were observed, demonstrating an effective energy transfer from the host to the emitters. Since the **BSQ-DMAC**-based OLED devices achieved the best EL performance, with a maximum luminance (L_max_) of 6042 cd m^−2^, maximum power efficiency (PE_max_) of 9.95 lm W^−1^ and maximum current efficiency (CE_max_) of 9.50 cd A^−1^, we further optimized the structure of this device by changing the dopant concentration. Finally, the highest EQE of 4.94% was obtained with an emission peak of 492 nm and an L_max_ of 7761 cd m^−2^ (Figure S17 and Table S1).

## 3. Conclusion

In conclusion, we designed and synthesized a series of sky-blue emitters, namely **BBI-4MeCz**, **BBI-DMAC**, **BSQ-4MeCz** and **BSQ-DMAC** based on novel polycyclic fused amide acceptor units. They all possessed orthogonal configurations between the D/A and π-spacers, forming a spatial steric effect. Moreover, the structure–property relationships of these four materials were studied completely with quantum computation, thermal analysis and photophysical characterization, and device performance was also assessed by fabricating OLED devices. As a result, due to the higher PLQY and *K*_F_, the photophysical properties and EL performance of the BSQ-based materials were generally superior to those of the BBI-based compounds. Eventually, the **BSQ-DMAC**-based device exhibited the highest EQE of 4.94% and a maximum luminance of 7761 cd m^−2^, with an emission peak of 492 nm. These findings demonstrate that polycyclic fused amide units can be a suitable candidate for the construction of blue emission materials due to their intrinsic features of a rigid planar structure and a high PLQY.

## Figures and Tables

**Table 1:** Key photophysical data of the as-synthesized materials.

Compound	*λ*_abs_[a]	*λ*_PL_[b]	*Φ*_f_ [c]	*τ*[d]	*E*_T1_/*E*_S1_[e]	HOMO/LUMO[f]	*E*_g_[g]	*T*_g/_*T*_d_[h]
	[nm]	[nm]	[%]	[ns]	[eV]	[eV]	[eV]	[°C]
**BBI-4MeCz**	334/348/409/427	482/512	9.1	0.8/6.9	1.73/2.62	−5.87/−3.14	2.73	137/408
**BBI-DMAC**	409/427	481/533	7.6	1.1/24.4	1.73/2.46	−5.77/−3.04	2.73	131/419
**BSQ-4MeCz**	392/413/438	474/484	77.3	4.6/6.3	1.81/2.74	−5.93/−3.17	2.76	n.d./463
**BSQ-DMAC**	392/413/438	474/517	78.1	5.1/12.6	1.81/2.56	−5.72/−2.96	2.76	126/430

[a] Absorption peaks in 10^−5^ M toluene solution; [b] PL peaks in 10^−5^ M toluene solution and thin film formed by spin-coating; [c] Absolute fluorescence quantum yields detected in toluene solution; [d] PL lifetimes in dilute toluene solution and thin film; [e] S_1_/T_1_ energy levels calculated based on B3LYP/6-31G(d,p); [f] HOMO level obtained from atmospheric ultraviolet photoelectron spectroscopies, LUMO level calculated from the equation: LUMO = HOMO + *E*_g_; [g] Optical energy gap; [h] Glass transition temperature and decomposition temperature (5% weight loss), n.d. means not detected.

**Table 2:** EL performances of the fabricated devices.

Emitter	*V*_on_ [a]	*λ*_EL_	*L*_max_	CE[b]	PE[b]	EQE[b]	CIE[c]
	[V]	[nm]	[cd m^−2^]	[cd A^−1^]	[lm W^−1^]	[%]	[x, y]
**mCP: 10 wt% BBI-4MeCz**	3.0	494	1703	7.54	7.88	2.95	(0.19, 0.40)
**mCP: 10 wt% BBI-DMAC**	3.2	494	2230	7.34	7.21	2.97	(0.18, 0.40)
**mCP: 10 wt% BSQ-4MeCz**	3.0	488	6187	8.58	8.90	4.13	(0.16, 0.31)
**mCP: 10 wt% BSQ-DMAC**	3.0	488	6042	9.50	9.95	4.45	(0.16, 0.32)

[a] Turn on voltage at 1 cd m^−2^; [b] maximum current efficiency (CE), power efficiency (PE), and external quantum efficiency (EQE); [c] Commission Internationale de l’Eclairage (CIE) coordinates at 6 V.

## Data Availability

Data is contained within the article or Supplementary Materials.

## Supplementary Materials

The following are available online at https://www.mdpi.com/article/10.3390/molecules27165181/s1 [33], Figure S1: 1H-NMR spectrum of compound BSQ-Br in deuterated CDCl3 solvent, Figure S2: 1H-NMR spectrum of compound BSQ-4MeCz in deuterated CDCl3 solvent, Figure S3: 13C-NMR spectrum of compound BSQ-4MeCz in deuterated CDCl3 solvent, Figure S4: 1H-NMR spectrum of compound BSQ-DMAC in deuterated CDCl3 solvent, Figure S5: 13C-NMR spectrum of compound BSQ-DMAC in deuterated CDCl3 solvent, Figure S6: 1H-NMR spectrum of compound BBI-4MeCz in deuterated CDCl3 solvent, Figure S7: 13C-NMR spectrum of compound BBI-4MeCz in deuterated CDCl3 solvent, Figure S8: 1H-NMR spectrum of compound BBI-DMAC in deuterated CDCl3 solvent, Figure S9: 13C-NMR spectrum of compound BBI-DMAC in deuterated CDCl3 solvent, Figure S10: The optimized ground state geometries of the investigated emitters, Figure S11: The natural transition orbitals (NTOs) distribution of S1 of the four emitters, Figure S12: The natural transition orbitals (NTOs) distribution of T1 of the four emitters, Figure S13: (a) TGA and (b) DSC curves of the as-synthesized materials, Figure S14: Atmospheric ultraviolet photoelectron spectroscopies of (a) BBI-4MeCz, (b) BBI-DMAC, (c) BSQ-4MeCz and (d) BSQ-DMAC, Figure S15: The PL spectra of (a) BSQ-4MeCz, (b) BSQ-DMAC, (d) BBI-4MeCz, (e) BBI-DMAC, (c) BSQ-Br and (f) BBI-Br in different solvents, Figure S16: The fluorescence and phosphorescence PL spectra of BSQ-4MeCz, BSQ-DMAC, BBI-4MeCz and BBI-DMAC at 77K, Figure S17: (a) EQE-L and (b) J-V-L characteristics of the optimized device based on BSQ-DMAC. (c) EL spectra of the optimized device based on BSQ-DMAC in different voltages. Table S1: The key data of optimized blue devices based on BSQ-DMAC.

## Sample Availability

Samples of the compounds are not available from the authors.
