# Supplementary material for: Novel Polycyclic Fused Amide Derivatives: Properties and Applications for Sky-blue Electroluminescent Devices
Source: Molecules. 2022 Aug 14;27(16):5181. doi: 10.3390/molecules27165181 (PMC9414544; doi:10.3390/molecules27165181)
Supplement: Supplementary file 1 [file molecules-27-05181-s001.zip › molecules-1864582-supplementary.pdf]

Supplementary Materials

Novel Polycyclic Fused Amide Derivatives: Synthesis, Properties and Applications for Blue Electroluminescent Devices

Guo-Xi Yang, Deng-Hui Liu, Si-Min Jiang, Zhi-Hai Yang, Zi-Jian Chen, Wei-Dong Qiu, Yi-Yang Gan, Kun-Kun Liu, De-Li Li and Shi-Jian Su**

State Key Laboratory of Luminescent Materials and Devices and Institute of Polymer Optoelectronic Materials and Devices, South China University of Technology, Wushan Road 381, Guangzhou 510640, P. R. China

Corresponding author: Dr. De-Li Li and Prof. Shi-Jian Su

E-mail: lideli@scut.edu.cn, mssjsu@scut.edu.cn

Contents

1. **General information**

2. **Device fabrication and characterization**

3. **Synthesis**

4. **Supplemental Figures and Tables**

5. **References**

1. General information

^{1}H-NMR and ^{13}C-NMR spectra were measured on Bruker NMR spectrometer (Germany) operating at 400/500 and 101/126 MHz, respectively, with tetramethylsilane (TMS) as the internal standard. All the data were recorded by dissolving the samples in deuterated chloroform ($CDCl_3$) solvent and measured at room temperature. Matrix-assisted laser desorption/ionization-time of flight (MALDI-TOF) mass spectrum measurement was obtained using an AXIMA-CFRTM plus instrument for confirming the molecular weight. Thermogravimetry (TGA) measurement was performed on Netzsch TG 209 at a heating rate of 10 ℃/min under a nitrogen flow atmosphere. Differential scanning calorimetry (DSC) analysis was obtained on Netzsch DSC 209 under a nitrogen flow atmosphere at three heating and cooling cycles with a heating rate of 10 ℃/min and a cooling rate of 20 ℃/min, respectively. The IP values were measured by atmospheric ultraviolet photoelectron spectroscopy (Rikken Keiki AC-3). Ultra-violet to visible (UV-vis) absorption spectra were performed using Perkin-Elmer Lambda 950-PKA, while photoluminescence (PL) spectra were recorded by FluoroMax-4 spectrofluorometer (Horiba Jobin Yvon). Photoluminescence quantum yields (PLQYs) of toluene solution were measured by utilizing a quantum efficiency measurement system of QE-2100. Transient PL decay was evaluated with 295 nm LED excitation source using EDINBURGH FLS980 system. All quantum chemical calculations were performed using the Gaussian 16 program package[1].

2. Device fabrication and characterization

Glass substrates pre-coated with a 95 nm thin layer of indium tin oxide (ITO) with a sheet resistance of 10 Ω per square were thoroughly cleaned in an ultrasonic bath of tetrahydrofuran (THF), isopropyl alcohol (IPA), detergent, deionized water, and isopropyl alcohol (IPA) and treated with O_2 plasma for 10 min in sequence. Organic layers were deposited onto the ITO-coated glass substrates by thermal evaporation under high vacuum (~10^{-5} Pa). Cathode, consisting of a 1 nm thin layer of LiF followed by a 100 nm thin Al layer, was patterned using a shadow mask with an array of 3 mm × 3 mm openings. Deposition rates are 1-2 Å s^{-1} for organic materials, 0.1 Å s^{-1} for LiF,

and 6 Å s^{-1} for Al, respectively. Electroluminescence (EL) spectra and CIE coordinates were recorded by an optical analyzer, Photo Research PR745. Current density and luminance versus driving voltage characteristics were measured by Keithley 2420 and Konica Minolta chromameter CS-200. External quantum efficiency (EQE) was calculated from the luminance, current density, and EL spectrum, assuming a Lambertian distribution. All the measurements were performed under ambient conditions at room temperature.

3. Synthesis

All solvents and reagents were purchased from commercial sources and used as received without further purification. The synthetic information of the targe compounds is outlined as below.

Synthetic routes of intermediates and target compounds

Intermediate compound **1** (8-iodonaphthalen-1-amine) was obtained from naphthalene-1,8-diamine according to the previously reported works.[2]

Synthesis of intermediate compound 2 (N-(8-iodonaphthalen-1-yl)benzamide)

8-Iodonaphthalen-1-amine (1.34 g, 4.98 mmol) and pyridine (0.55 g, 6.97 mmol) were added into dry dichloromethane (30 mL) at 0 °C. Then benzoyl chloride (0.84 g, 5.98 mmol) was added slowly into the solution. After being stirred at room temperature overnight, the solvent was removed directly by rotary evaporation and purified by silica gel column chromatography to afford the desired compound as a white solid in 86.0% yield.

Synthesis of intermediate compound 3 (N-(8-((4-bromophenyl)ethynyl)naphthalen-1-yl)benzamide)

Under nitrogen atmosphere, compound **2** (16.0 g, 42.8 mmol), 1-bromo-4-ethynylbenzene (9.3 g, 51.3 mmol), cuprous iodide (0.245 g, 3 mol%) and palladium catalyst (0.90 g, 3 mol%) were added to a solution of triethylamine (400 mL), and the mixture was stirred at room temperature for at least 12 hours. Then, water (1000 mL)

was added, the mixed solution was extracted with dichloromethane (3 × 500 mL) and concentrated by rotary evaporation. Further purification by column chromatography on silica gel to give the desired compound as pale-yellow solids in 57.8% yield.

Synthesis of intermediate compound 4 (8-((4-bromophenyl)ethynyl)naphthalen-1-amine)

The synthetic route of compound **4** is the same as intermediate compound **3**. After purification by column chromatography on silica gel, a pale-yellow solid was acquired with yield of 63.6%.

Synthesis of intermediate compound 5 (N-(8-((4-bromophenyl)ethynyl)naphthalen-1-yl)benzenesulfonamide)

The synthetic route of compound **5** is the same as intermediate compound **2**. After purification by column chromatography on silica gel, a pale-yellow solid was acquired with yield of 81.2%.

Synthesis of BSQ-Br (13-(4-bromophenyl)-8H-benzo[3,4]indolo[1,2-b]isoquinolin-8-one)

Compound **3** (5.4 g, 12.67 mmol) and cuprous cyanide (0.22 g, 0.2 eq) were added into a 150 mL three-necked flask, then DMF (60 mL) was added. The mixture was stirred at 110 °C (oil bath, checked by TLC). After compound **3** was completely consumed, the reaction mixture was then cooled to room temperature and quenched by adding 200 mL of water and extracted with dichloromethane. After the organic layer was washed with saturated salt water, it was filtrated and concentrated under reduced pressure. The residue was purified by column chromatography to afford the desired products BSQ-Br as a yellow solid in 20.5% yield. ^{1}H NMR (500 MHz, Chloroform-d) δ 8.70 – 8.64 (m, 1H), 8.58 – 8.50 (m, 1H), 7.85 – 7.77 (m, 3H), 7.71 – 7.59 (m, 3H), 7.58 – 7.52 (m, 1H), 7.45 – 7.35 (m, 3H), 7.32 – 7.22 (m, 1H), 6.66 – 6.59 (m, 1H).

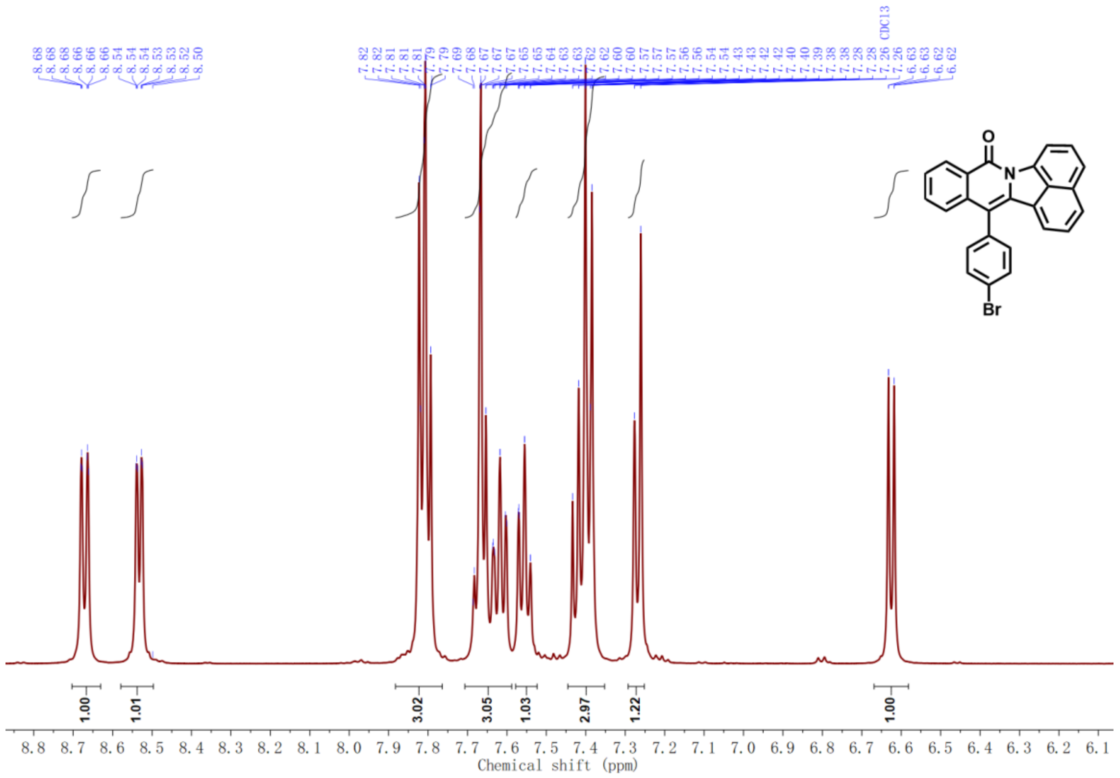

Figure S1. ^{1}H-NMR spectrum of compound BSQ-Br in deuterated $CDCl_3$ solvent.

Synthesis of BBI-Br (13-(4-bromophenyl)benzo[cd]benzo[5,6][1,2]thiazino[2,3-a]indole 8,8-dioxide)

Compound **5** (5.0 g, 10.8 mmol), cuprous iodide (1.0 g, 0.5 eq) and *tert*-butyl hydroperoxide (2.0 g, 2 eq) were added into a 150 mL three-necked flask and then DMSO (60 mL) was added. The mixture was stirred at 110 °C (oil bath, checked by TLC). After compound **5** was completely consumed, the reaction mixture was then cooled to room temperature and quenched by adding 200 mL of water and extracted with dichloromethane. After the organic layer was washed with saturated salt water, it was filtrated and concentrated under reduced pressure. The residue was purified by column chromatography to afford the desired product BBI-Br as a yellow solid in 33.0% yield.

Synthesis of BSQ-4MeCz (13-(4-(1,3,6,8-tetramethyl-9H-carbazol-9-yl)phenyl)-8H-benzo[3,4]indolo[1,2-b]isoquinolin-8-one)

Under nitrogen atmosphere, compound BSQ-Br (0.8 g, 1.9 mmol), 1,3,6,8-

tetramethyl-9H-carbazole (0.46 g, 2.1 mmol), $Pd(OAc)_2$ (21.2 mg, 5 mol%), tri-*tert*-butylphosphine tetrafluoroborate (54.5 mg, 10 mol%) and sodium *tert*-butoxide (0.91 g, 5 eq) were added to a solution of toluene (80 mL), and the mixture was stirred at 110 °C for at least 12 hours. After cooled down to ambient temperature, the resulting solution was extracted with dichloromethane. The concentrated organic layer was evaporated and purified by column chromatography with a mixture eluent of dichloromethane/petroleum ether, resulting in a yellow powder (0.85 g) in 79.6% yield. ^{1}H NMR (400 MHz, Chloroform-d) δ (ppm) 8.74 (d, J = 7.8 Hz, 1H), 8.59 (dd, J = 5.8, 2.1 Hz, 1H), 7.86 (d, J = 8.1 Hz, 1H), 7.81 (t, J = 4.0 Hz, 4H), 7.74 – 7.66 (m, 3H), 7.65 – 7.57 (m, 3H), 7.41 (t, J = 7.7 Hz, 1H), 7.32 (d, J = 8.0 Hz, 1H), 7.02 (s, 2H), 6.95 (d, J = 7.3 Hz, 1H), 2.52 (s, 6H), 2.33 (d, J = 28.2 Hz, 6H). ^{13}C NMR (101 MHz, Chloroform-d) δ (ppm) 143.17, 139.53, 138.06, 136.77, 136.03, 135.41, 132.98, 132.70, 130.77, 130.36, 129.74, 129.34, 129.28, 128.40, 128.22, 127.34, 126.77, 125.84, 125.21, 124.37, 122.58, 121.44, 118.05, 117.27, 113.80, 21.16. MS (ESI) (m/z): Calculated for $C_{41}H_{30}N_2O$: 566.70 Found m/z (MALDI-TOF): 566.1915.

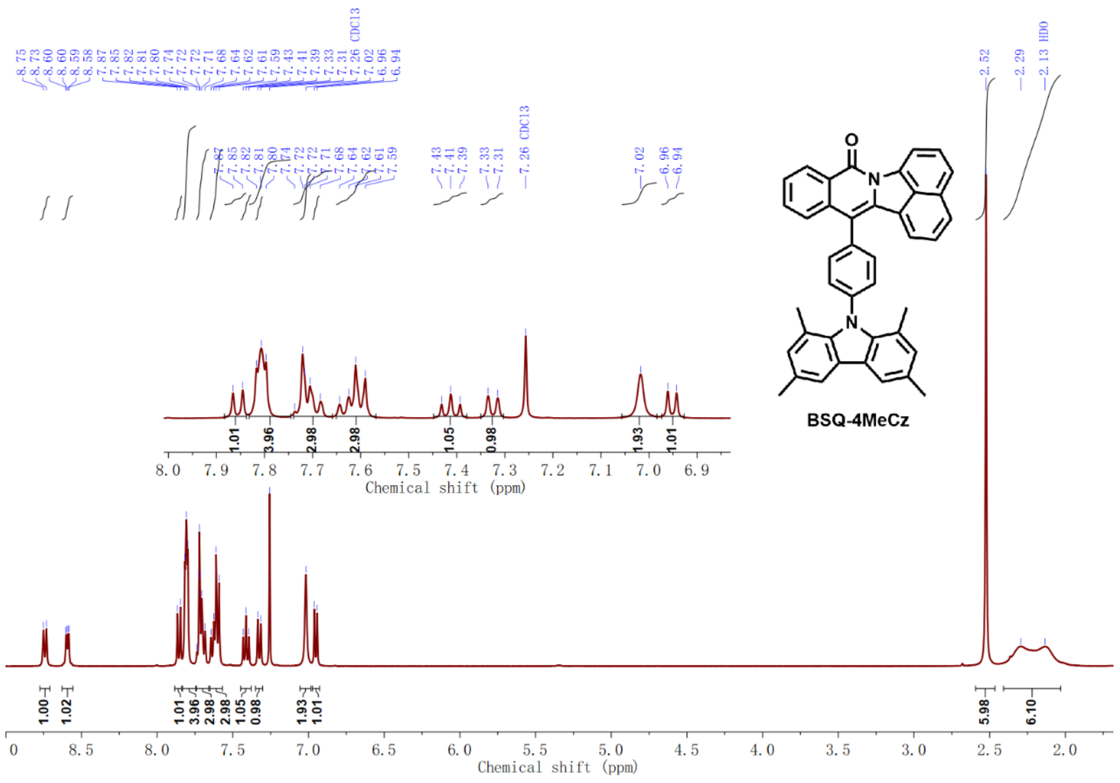

Figure S2. ^{1}H-NMR spectrum of compound BSQ-4MeCz in deuterated $CDCl_3$ solvent.

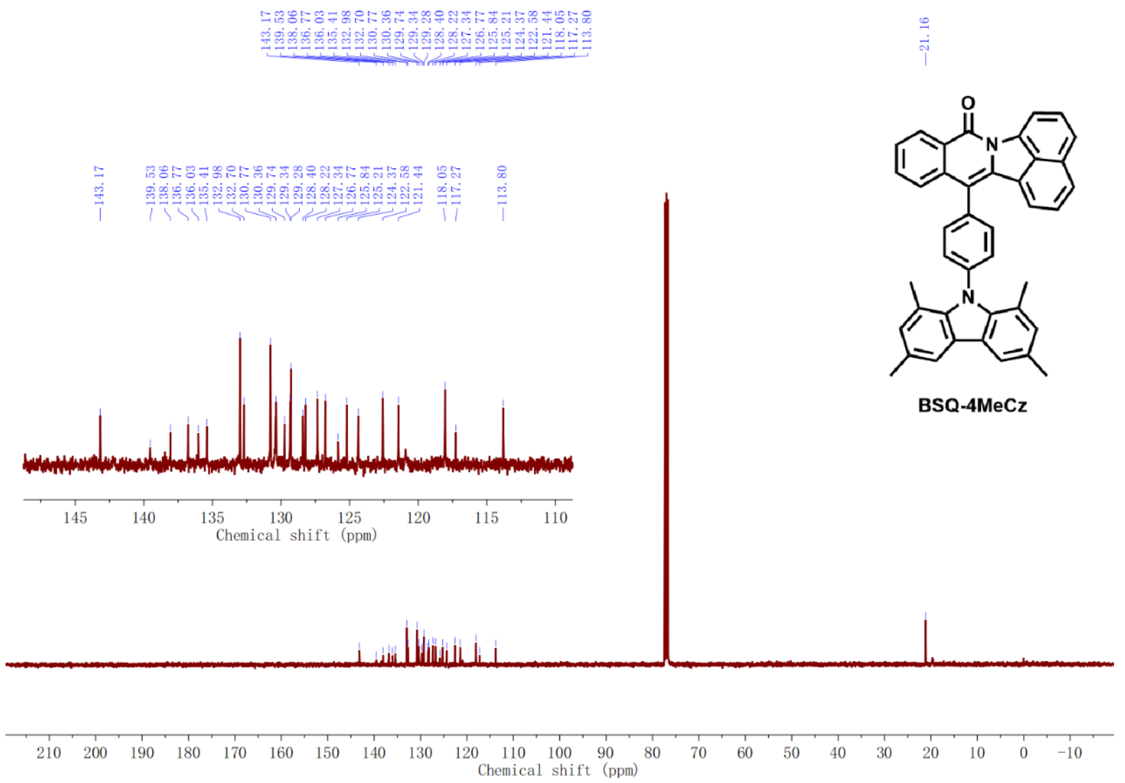

Figure S3. ^{13}C-NMR spectrum of compound BSQ-4MeCz in deuterated $CDCl_3$ solvent.

Synthesis of BSQ-DMAC (13-(4-(9,9-dimethylacridin-10(9H)-yl)phenyl)-8H-benzo[3,4]indolo[1,2-b]isoquinolin-8-one)

The synthetic route of the compound BSQ-DMAC is the same as the compound BSQ-4MeCz except for replacing compound 1,3,6,8-tetramethyl-9H-carbazole with 9,9-dimethyl-9,10- dihydroacridine (0.54 g, 2.6 mmol), resulting in a yellow solid (0.85 g) in 65.3% yield. ^{1}H NMR (500 MHz, Chloroform-d) δ (ppm) 8.74 (dd, *J* = 8.1, 1.4 Hz, 1H), 8.59 (dd, *J* = 6.1, 1.9 Hz, 1H), 7.85 (d, *J* = 8.1 Hz, 1H), 7.80 – 7.61 (m, 8H), 7.56 – 7.43 (m, 4H), 7.14 (ddd, *J* = 8.3, 7.1, 1.6 Hz, 2H), 7.02 (td, *J* = 7.5, 1.3 Hz, 2H), 6.79 (d, *J* = 7.2 Hz, 1H), 6.57 (dd, *J* = 8.2, 1.2 Hz, 2H), 1.76 (s, 6H). ^{13}C NMR (101 MHz, Chloroform-d) δ (ppm) 160.43, 141.71, 140.83, 138.09, 136.74, 136.23, 135.03, 133.25, 133.09, 132.64, 130.45, 130.33, 129.78, 129.30, 128.45, 128.35, 127.36, 127.31, 126.73, 126.46, 125.80, 125.61, 125.38, 122.54, 121.27, 120.95, 117.51, 113.99, 113.75, 77.21, 36.11, 31.04. MS (ESI) (m/z): Calculated for $C_{40}H_{28}N_2O$: 552.68.

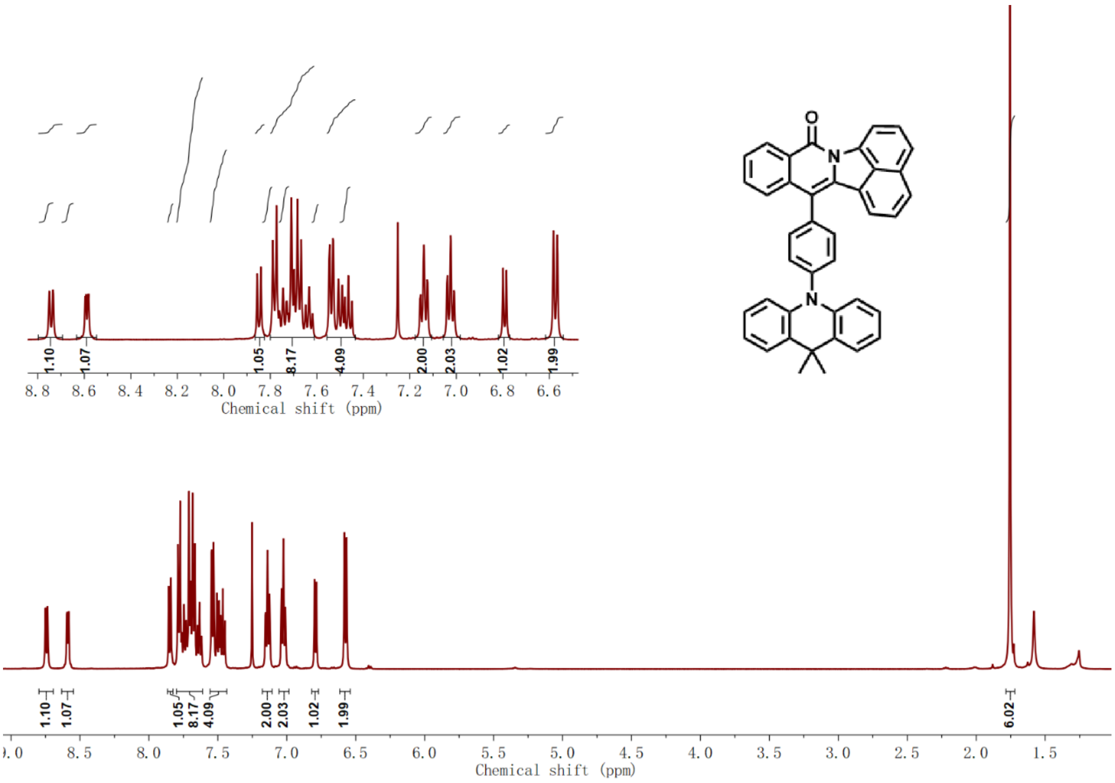

Figure S4. ^{1}H-NMR spectrum of compound BSQ-DMAC in deuterated $CDCl_3$ solvent.

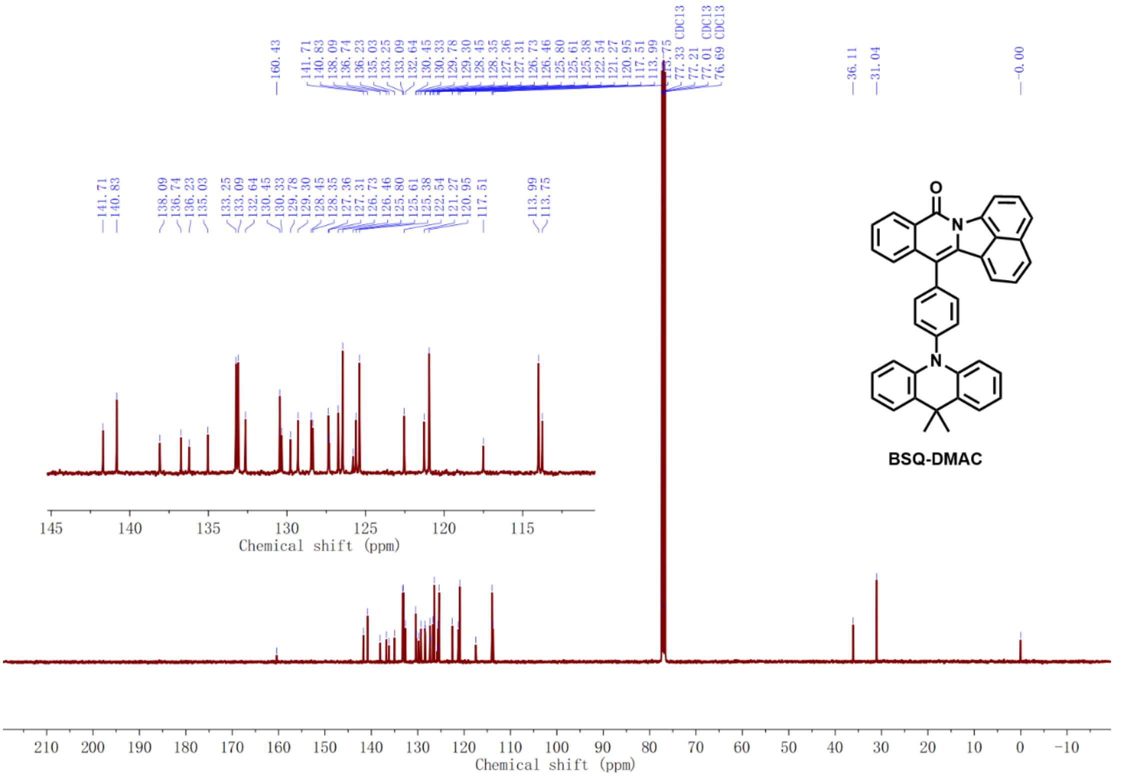

Figure S5. ^{13}C-NMR spectrum of compound BSQ-DMAC in deuterated $CDCl_3$ solvent.

Synthesis of BBI-4MeCz (13-(4-(1,3,6,8-tetramethyl-9H-carbazol-9-

yl)phenyl)benzo[cd]benzo[5,6][1,2]thiazino[2,3-a]indole 8,8-dioxide)

Under nitrogen atmosphere, compound BBI-Br (1.1 g, 2.4 mmol), 1,3,6,8-tetramethyl-9H-carbazole (0.59 g, 2.6 mmol), $Pd(OAc)_2$ (26.8 mg, 5 mol%), tri-*tert*-butylphosphine tetrafluoroborate (69.1 mg, 10 mol%) and sodium *tert*-butoxide (1.15 g, 5 eq) were added to a solution of toluene (80 mL), and the mixture was stirred at 110 °C for at least 12 hours. After cooled down to ambient temperature, the resulting solution was extracted with dichloromethane. The concentrated organic layer was evaporated and purified by column chromatography with a mixture eluent of dichloromethane/petroleum ether, resulting in a yellow powder (0.90 g) in 62.5% yield. ^{1}H NMR (500 MHz, Chloroform-d) δ (ppm) 8.23 (dd, J = 7.7, 1.5 Hz, 1H), 7.86 – 7.74 (m, 6H), 7.64 – 7.53 (m, 6H), 7.33 (t, J = 7.7 Hz, 1H), 7.13 (dd, J = 7.9, 1.2 Hz, 1H), 7.00 (s, 2H), 6.72 (d, J = 7.3 Hz, 1H), 2.52 (s, 6H), 2.07 (s, 6H). ^{13}C NMR (126 MHz, Chloroform-d) δ (ppm) 143.41, 138.84, 137.22, 135.11, 134.49, 133.31, 132.75, 131.26, 131.04, 130.84, 130.82, 130.39, 129.31, 129.03, 128.49, 128.02, 127.63, 127.11, 126.23, 124.38, 122.87, 121.90, 121.25, 118.04, 116.51, 109.63, 21.13. MS (ESI) (m/z): Calculated for $C_{40}H_{30}N_2O_2S$: 602.75 Found m/z (MALDI-TOF): 602.1470.

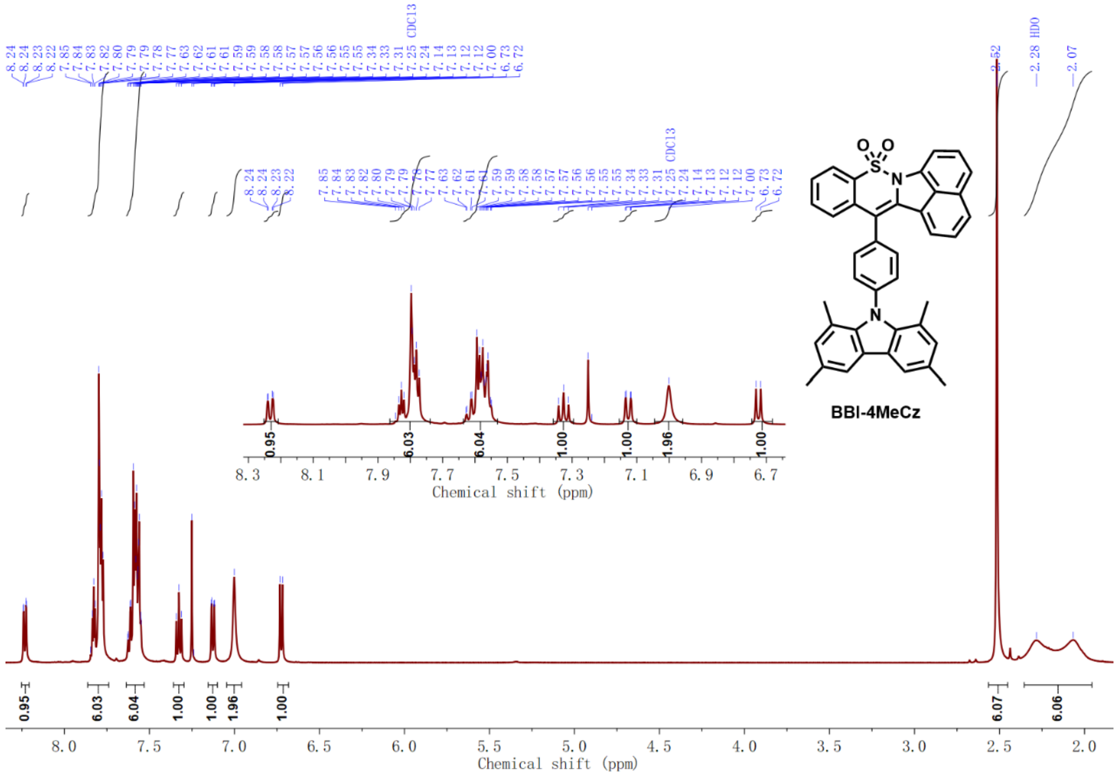

Figure S6. ^{1}H-NMR spectrum of compound BBI-4MeCz in deuterated $CDCl_3$ solvent.

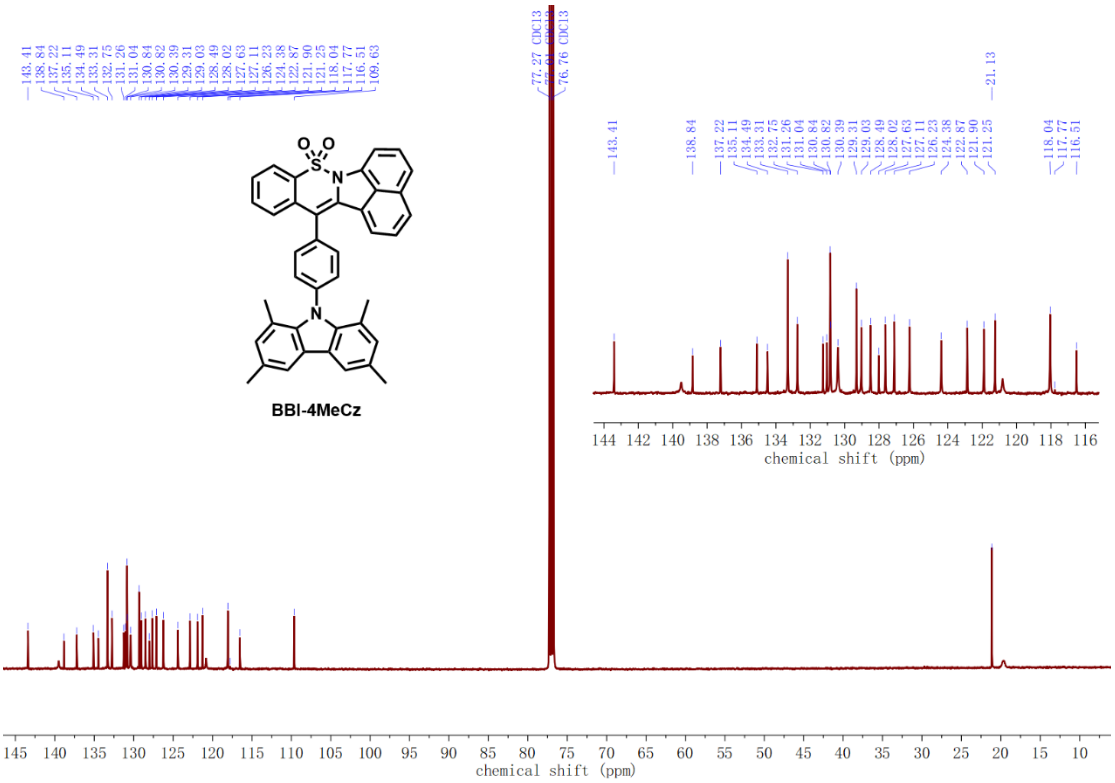

Figure S7. ^{13}C-NMR spectrum of compound BBI-4MeCz in deuterated $CDCl_3$ solvent.

Synthesis of BBI-DMAC (13-(4-(9,9-dimethylacridin-10(9H)-yl)phenyl)benzo[cd]benzo[5,6][1,2]thiazino[2,3-a]indole 8,8-dioxide)

The synthetic route of the compound BBI-DMAC is the same as compound BBI-4MeCz except for replacing compound 1,3,6,8-tetramethyl-9H-carbazole with 9,9-dimethyl-9,10-dihydroacridine (0.55 g, 2.6 mmol), resulting in a yellow solid (0.80 g) in 56.9% yield. 1H NMR (500 MHz, Chloroform-d) δ (ppm) 8.25 (d, J = 7.8 Hz, 1H), 7.83 (q, J = 5.1, 4.6 Hz, 1H), 7.77 (dd, J = 17.5, 8.1 Hz, 3H), 7.70 – 7.64 (m, 3H), 7.60 (d, J = 4.2 Hz, 3H), 7.55 – 7.51 (m, 2H), 7.39 (t, J = 7.7 Hz, 1H), 7.32 (d, J = 8.0 Hz, 1H), 7.16 – 7.09 (m, 2H), 7.02 (t, J = 7.4 Hz, 2H), 6.53 (dd, J = 12.6, 7.7 Hz, 3H), 1.75 (s, 6H). ^{13}C NMR (126 MHz, Chloroform-d) δ (ppm) 141.94, 140.75, 139.05, 137.29, 134.77, 134.42, 133.49, 133.32, 132.75, 131.20, 131.03, 130.90, 130.51, 129.02, 128.75, 128.01, 127.67, 127.09, 126.64, 126.44, 125.40, 122.86, 121.71, 121.22, 121.00, 116.73, 113.95, 109.58, 36.11, 31.01. MS (ESI) (m/z): Calculated for $C_{39}H_{28}N_2O_2S$: 588.73.

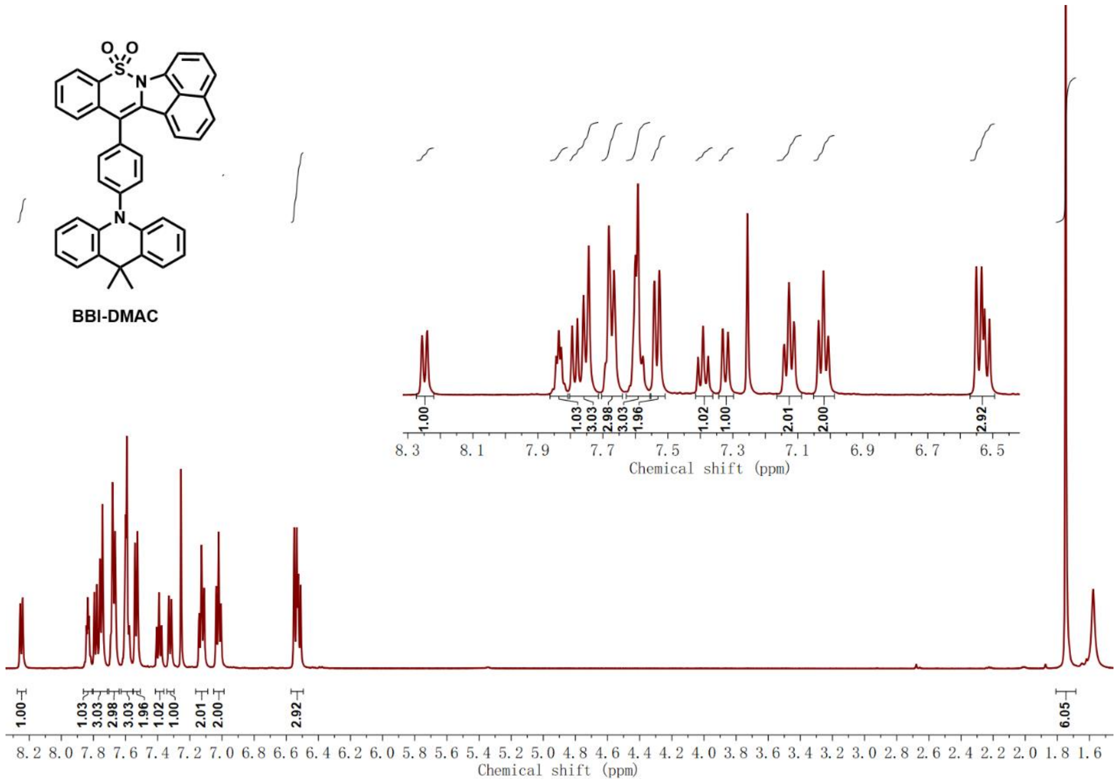

Figure S8. ¹H-NMR spectrum of compound BBI-DMAC in deuterated $CDCl_3$ solvent.

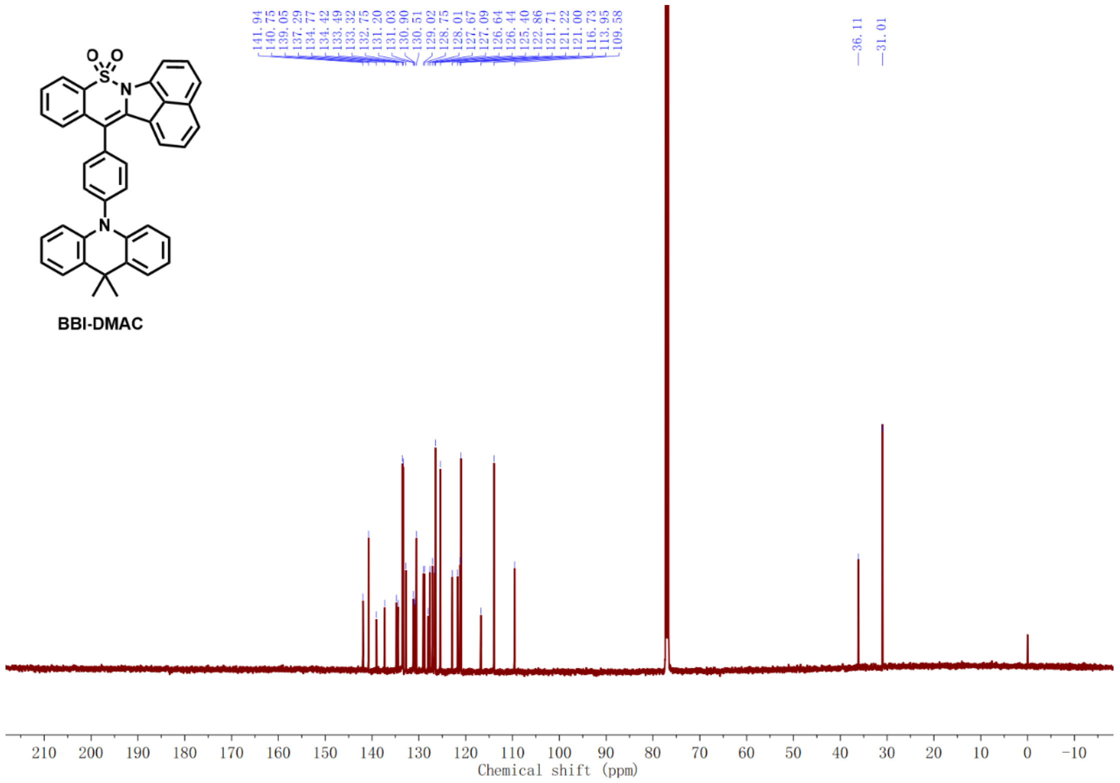

Figure S9. ¹³C-NMR spectrum of compound BBI-DMAC in deuterated $CDCl_3$ solvent.

4. Supplemental Figures and Tables

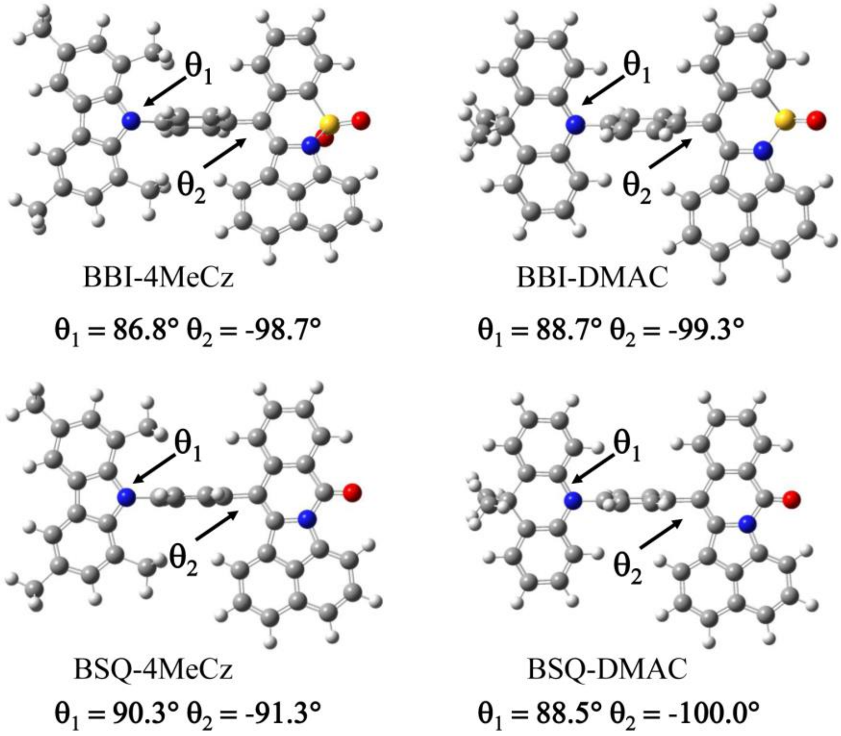

Figure S10. The optimized ground state geometries of the investigated emitters.

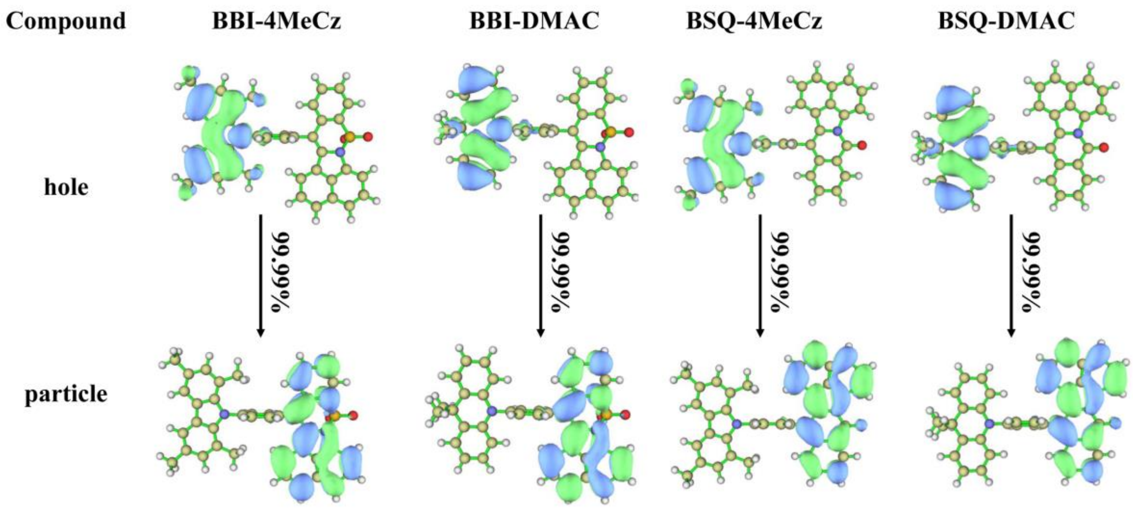

Figure S11. The natural transition orbitals (NTOs) distribution of S_1 of the four emitters.

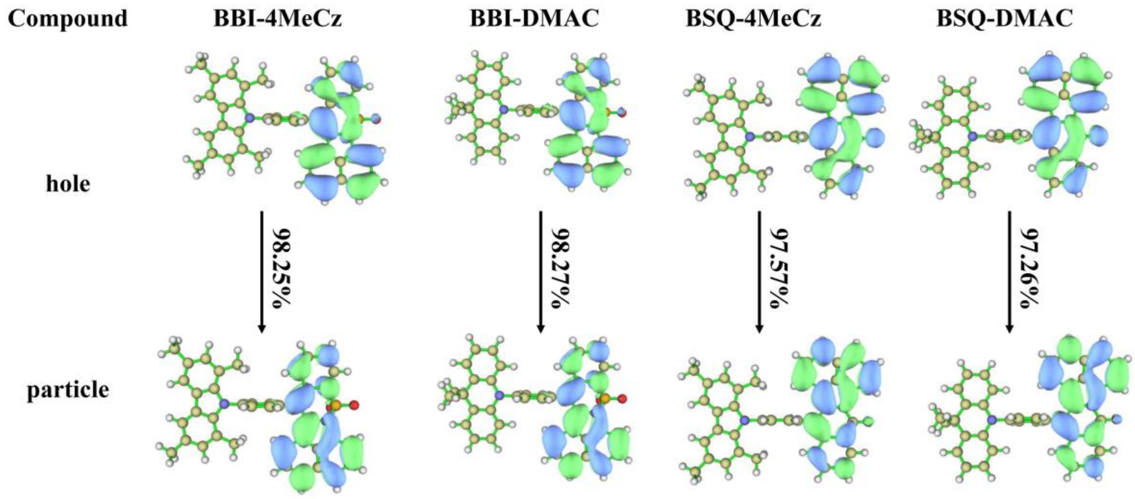

Figure S12. The natural transition orbitals (NTOs) distribution of T_1 of the four emitters.

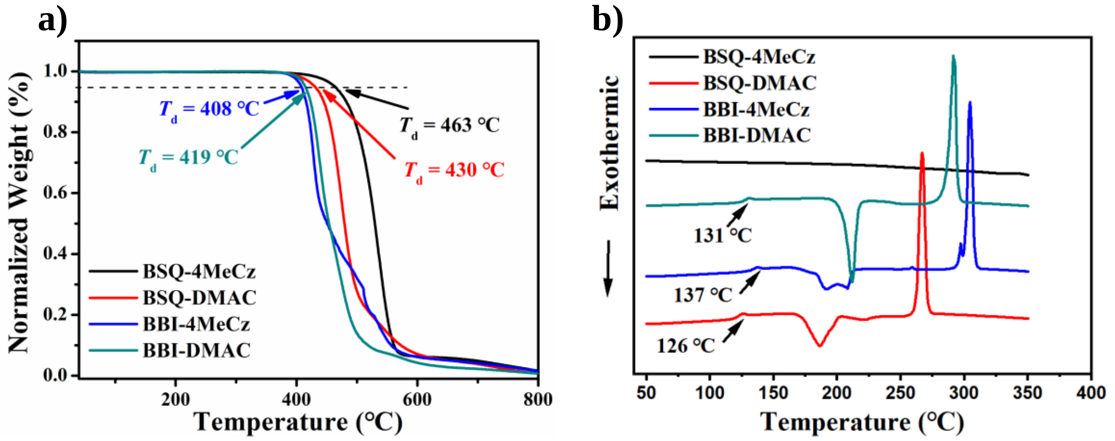

Figure S13. a) TGA and b) DSC curves of the as-synthesized materials.

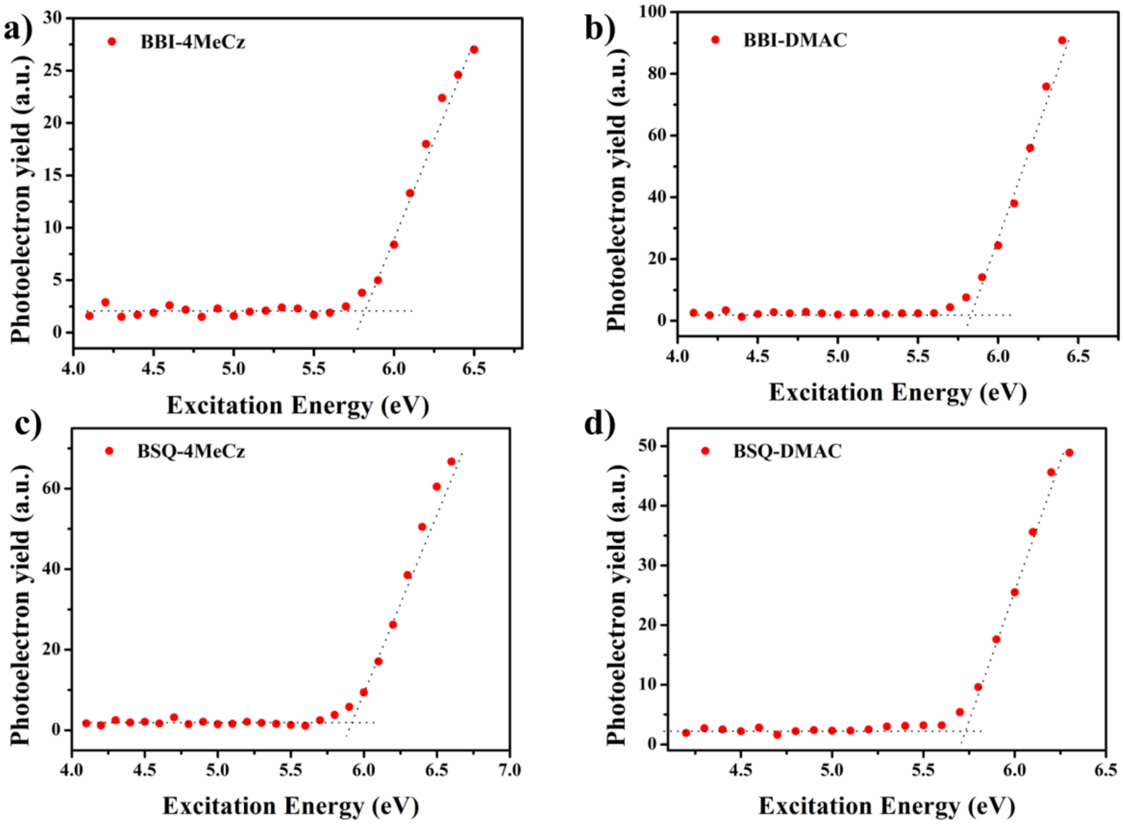

Figure S14. Atmospheric ultraviolet photoelectron spectroscopies of a) BBI-4MeCz, b) BBI-DMAC, c) BSQ-4MeCz and d) BSQ-DMAC.

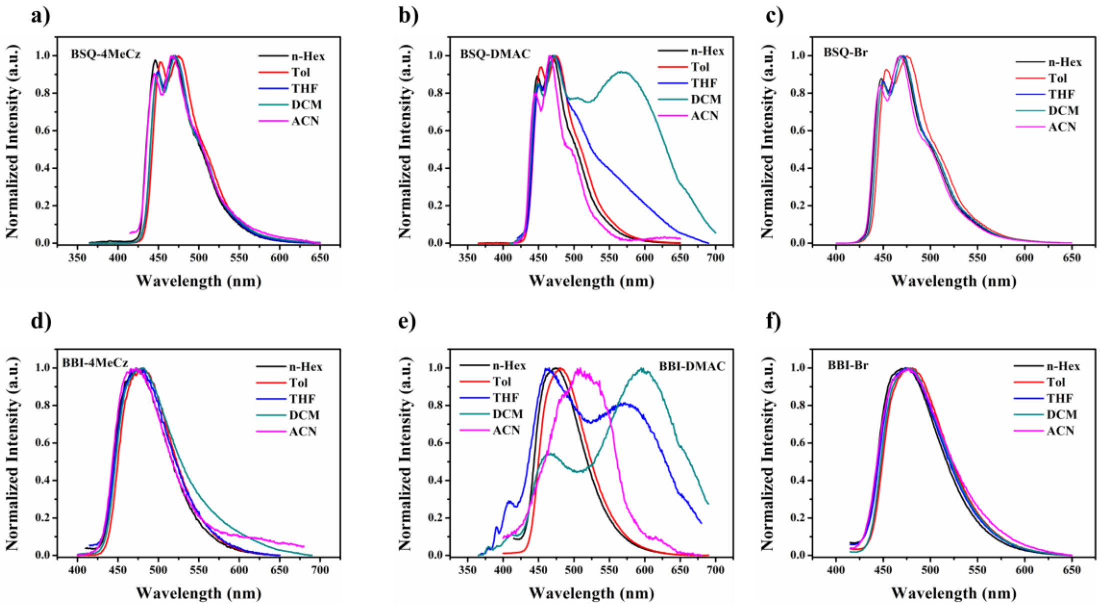

Figure S15. The PL spectra of a) BSQ-4MeCz, b) BSQ-DMAC, d) BBI-4MeCz, e) BBI-DMAC, c) BSQ-Br and f) BBI-Br in different solvents.

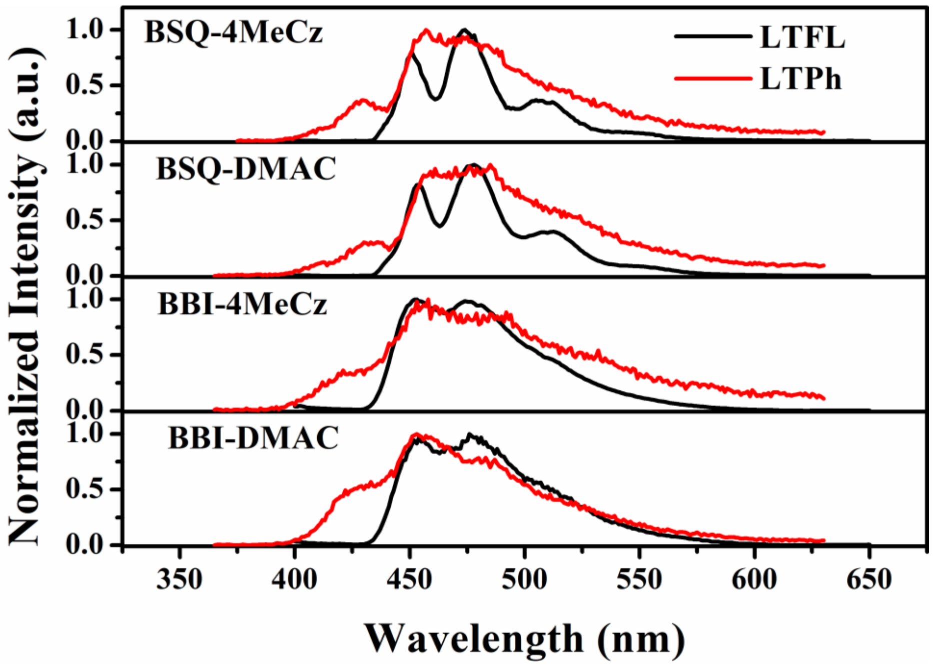

Figure S16. The fluorescence and phosphorescence PL spectra of BSQ-4MeCz, BSQ-DMAC, BBI-4MeCz and BBI-DMAC at 77K.

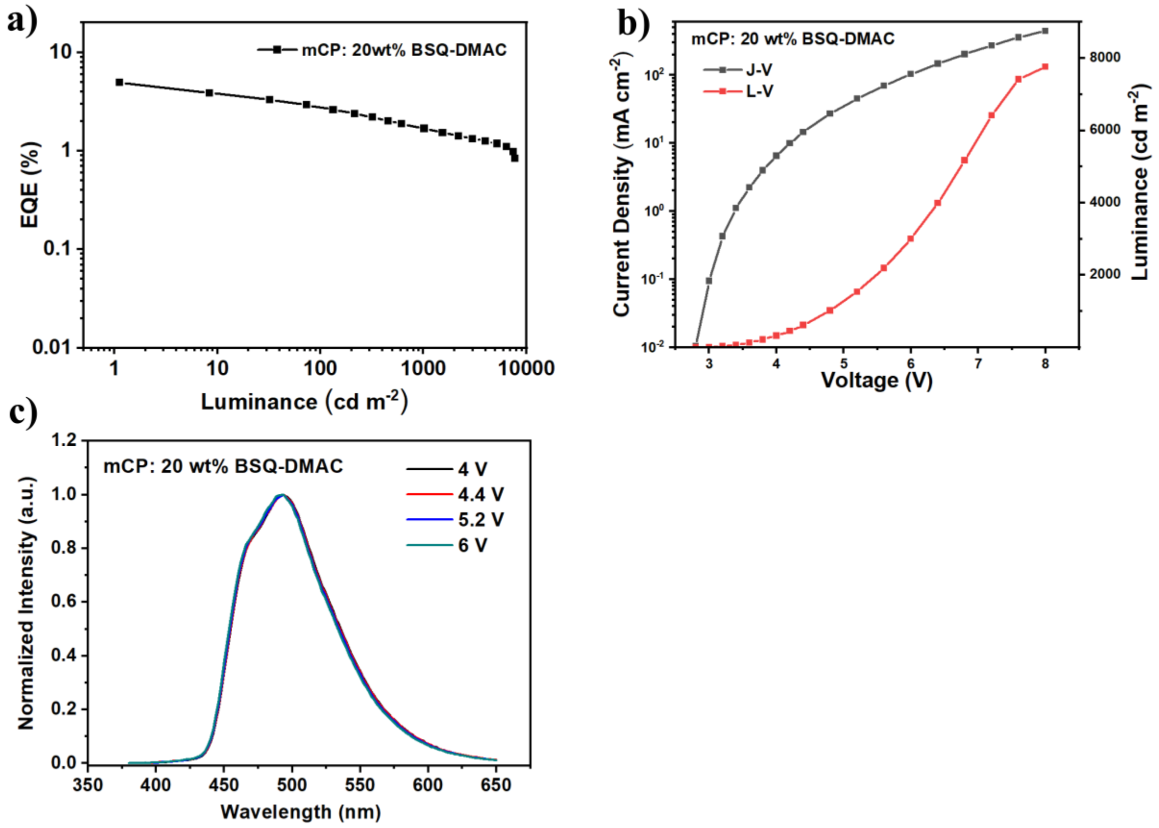

Figure S17. a) EQE-L and b) *J*-*V*-*L* characteristics of the optimized device based on BSQ-DMAC. c) EL spectra of the optimized device based on BSQ-DMAC in different voltages.

Table S1 The key data of optimized blue devices based on BSQ-DMAC

Emitter	V_{on} [a] [V]	λ_{EL} [nm]	L_{max} [cd m^{-2}]	CE[b] [cd A^{-1}]	PE[b] [lm W^{-1}]	EQE[b] [%]	CIE[c] [x, y]
mCP: 5 wt% BSQ-DMAC	3.1	488	5080	8.95	9.07	4.56	(0.15, 0.29)
mCP: 20 wt% BSQ-DMAC	2.8	492	7761	10.93	12.26	4.94	(0.17, 0.35)

[a] Turn on voltage at 1 cd m^{-2}; [b] maximum current efficiency (CE), power efficiency (PE), and external quantum efficiency (EQE), [c] Commission Internationale de l´Eclairage (CIE) coordinates at 4 V.

5. References

1. Li, W.; Li, W.; Gan, L.; Li, M.; Zheng, N.; Ning, C.; Chen, D.; Wu, Y. C.; Su, S. J., J-aggregation enhances the electroluminescence performance of a sky-blue thermally activated delayed-fluorescence emitter in nondoped organic light-emitting diodes. *ACS Appl. Mater. Interfaces* **2020,** *12*, 2717-2723.
2. Wang, B.; Daugulis, O.; Brookhart, M., Ethylene polymerization with Ni(II) diimine complexes generated from 8-halo-1-naphthylamines: The role of equilibrating Syn/Anti diastereomers in determining polymer properties. *Organometallics* **2019,** *38*, 4658-4668.
